# Structural degeneracy and formation of crystallographic domains in epitaxial LaFeO_3_ films revealed by machine-learning assisted 4D-STEM

**DOI:** 10.1038/s41598-024-54661-1

**Published:** 2024-02-20

**Authors:** Menglin Zhu, Joseph Lanier, Jose Flores, Victor da Cruz Pinha Barbosa, Daniel Russell, Becky Haight, Patrick M. Woodward, Fengyuan Yang, Jinwoo Hwang

**Affiliations:** 1 Department of Materials Science and Engineering, Ohio State University, Columbus, OH 43210 USA; 2 Department of Physics, Ohio State University, Columbus, OH 43210 USA; 3 Department of Chemistry and Biochemistry, Ohio State University, Columbus, OH 43210 USA

**Keywords:** Transmission electron microscopy, Structure of solids and liquids

## Abstract

Structural domains and domain walls, inherent in single crystalline perovskite oxides, can significantly influence the properties of the material and therefore must be considered as a vital part of the design of the epitaxial oxide thin films. We employ 4D-STEM combined with machine learning (ML) to comprehensively characterize domain structures at both high spatial resolution and over a significant spatial extent. Using orthorhombic LaFeO_3_ as a model system, we explore the application of unsupervised and supervised ML in domain mapping, which demonstrates robustness against experiment uncertainties. The results reveal the consequential formation of multiple domains due to the structural degeneracy when LaFeO_3_ film is grown on cubic SrTiO_3_. In situ annealing of the film shows the mechanism of domain coarsening that potentially links to phase transition of LaFeO_3_ at high temperatures. Moreover, synthesis of LaFeO_3_ on DyScO_3_ illustrates that a less symmetric orthorhombic substrate inhibits the formation of domain walls, thereby contributing to the mitigation of structural degeneracy. High fidelity of our approach also highlights the potential for the domain mapping of other complicated materials and thin films.

## Introduction

Advancements in epitaxial growth techniques have ushered in a new era for producing single crystalline oxide films with atomically sharp interfaces, opening up possibilities for achieving properties previously unattainable in bulk materials^1–4^. These systems have benefited from the structural homogeneity and crystallographic integrity. However, achieving such homogeneity can still be challenging in oxides with intricate structural complexities^5–7^.

One possible consequence of the interplay between lattice, charge, and other constraints is the emergence of crystallographic domains with specific symmetry relationships, such as mirror, inversion, or rotational symmetry operations, setting them apart from grain boundaries where two grains can meet at any angle^5–9^. Furthermore, the potential coupling between ferroelectric or magnetic axes with crystalline axes highlights the critical role that structural domains can play in altering the functional properties of epitaxial thin films^6,10–12^. As a result, the concept of domain engineering must be considered in the design of epitaxial thin films, which requires understanding of how structural domains form and behave with different external parameters, such as strain and temperature.

In perovskite oxides (ABO_3_), twin domains can readily form during growth or emerge via phase transformations afterward. The inherent propensity for domain formation paves the way for tailoring the materials’ properties. For example, precise control over domain formation allows for a broad spectrum of adjustments in the magnetic and transport properties of SrRuO_3_, owing to the interplay between the magnetic easy axis and the crystallographic axis^6^. In ferroelectric perovskites, spontaneous polarization may lead to the evolution of domains from simple shapes to more intricate structures, such as nanodomains and vortices^3,13–15^, offering new opportunities for device design. In addition, domain walls naturally exhibit reduced dimensionality and different symmetry from the host material, resulting in unique physical properties^5,16–19^.

Unlocking the functionalities of domains and domain walls presents a pivotal challenge that necessitates high spatial resolution characterization of domain structures across large scales. This enables the understanding of individual domain behaviors and their influence on global material properties. Scanning/transmission electron microscopy (S/TEM) offers unique advantages for this purpose, including high-resolution direct imaging of domain structures and other complementing techniques such as chemical composition analysis. Techniques like TEM dark field or STEM differential phase contrast (DPC) imaging have found extensive applications in revealing ferroelastic(electric) domains, for example, in BaTiO_3_ and LaCoO_3_ at nano scale^20–24^. However, prior knowledge of the materials' structure is often essential to set up the appropriate experimental condition, and interpretation of image contrast can be ambiguous. This ambiguity can be particularly challenging in cases with a high degree of freedom in domain orientation, where structural details may be lost during the imaging process. The advent of high-speed pixelated detectors enables the capture of diffraction patterns (DPs) at each scanning position, namely 4D-STEM^25,26^. These DPs collectively contain the abundant structural details of local regions, thus facilitating comprehensive analysis of domains. However, the sheer amount of data generated by 4D-STEM, akin to other hyperspectral data types like electron energy loss spectroscopy (EELS), poses a significant challenge for data analysis.

One of the promising approaches is to apply machine learning (ML) algorithms to identify patterns and correlations that may not be immediately apparent^27,28^. This approach can be categorized into two main paradigms: supervised and unsupervised learning. In supervised learning, algorithms are trained on labeled datasets, where the model learns from input–output pairs to make predictions on new, unseen data. Conversely, unsupervised learning operates without explicit output labels, allowing algorithms to explore patterns and structures within the input data independently. Both paradigms have demonstrated notable success in data-driven microscopy analyses, with supervised learning excelling in mimicking the intricate neural structure of the human brain and identifying features of interest from microscopy data^29,30^. While unsupervised learning ML has proven valuable in exploratory tasks, such as unveiling internal structure and denoising hyperspectral data^31–36^.

In this study, we explore the potential of ML-assisted analysis of 4D-STEM data to map crystallographic domains in epitaxial oxide films. Experimentally, the 4D-STEM DPs are acquired using a fast pixelated detector. The subsequent implementation of ML to the 4D data involves several key steps. First, we extract feature vectors (FVs) from each DP using an unsupervised approach (SimSiam-based contrastive learning)^37^. By clustering these FVs, we can distinguish domains related by a 90-degree rotation, effectively uncovering their distinct structural characteristics. Afterwards, we employ a supervised learning approach to further differentiate domains related by mirror symmetry, akin to ferroelectric domains with opposing polarization directions. This multifaceted approach showcases resilience against diverse experimental conditions and facilitates efficient mapping across a broader field of view, thereby enhancing the statistical significance of our results. Using LaFeO_3_ (LFO) as a model system, we show that (1) crystallographic domains in LFO films can originate from the structural degeneracy of the substrate/film, (2) certain orientations are favored to minimize elastic energy caused by lattice mismatches, (3) domains undergo a coarsening process at elevated temperatures during in-situ annealing, and (4) the degeneracy of structural domains can be mitigated by employing a substrate with in-plane anisotropy.

## Methods and results

### Formation of crystallographic domains in LFO

**Figure 1:**
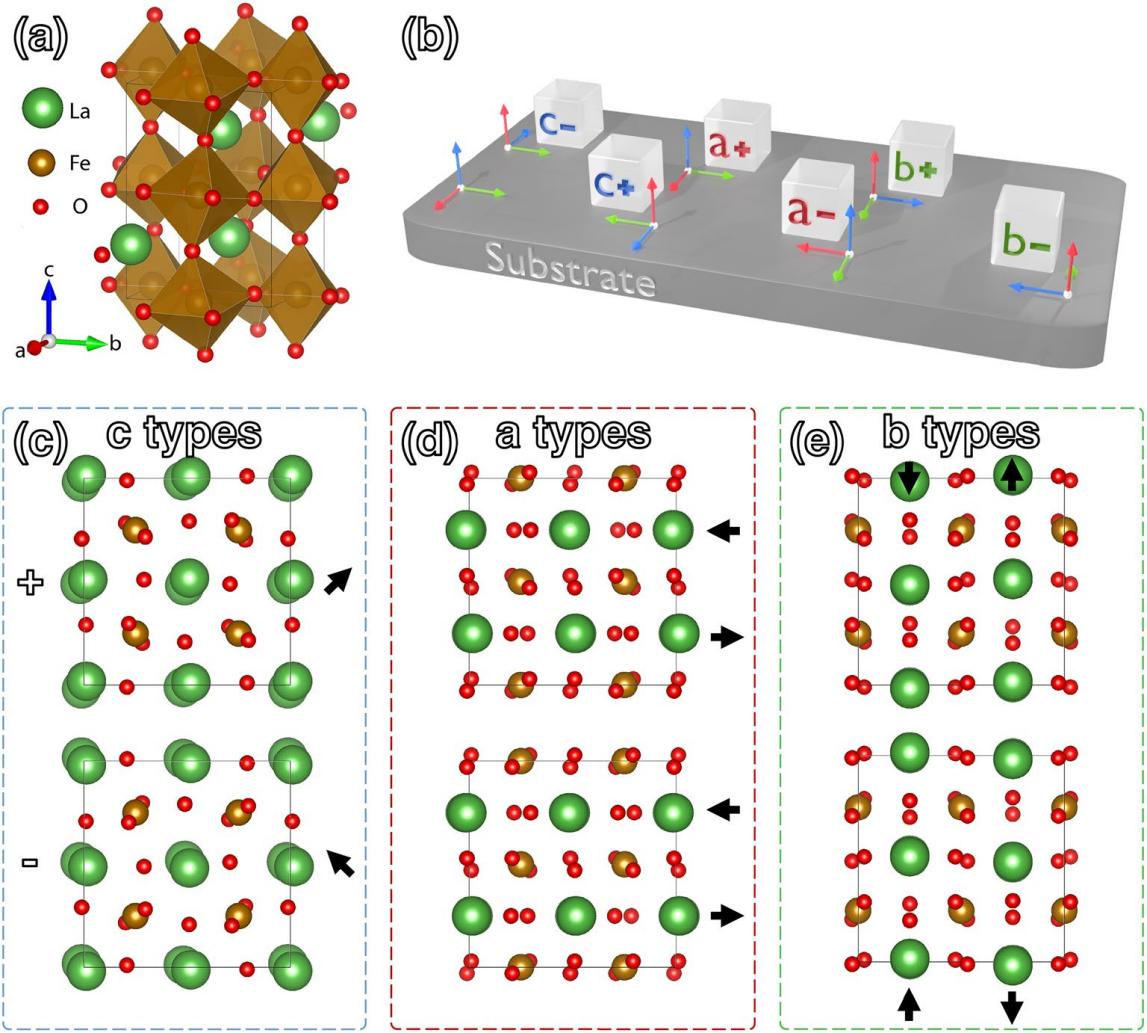
(**a**) Crystal model of LaFeO_3_ orthorhombic unit cell. (**b**) Six possible domain configurations on perovskite substrate with orthogonal coordinates corresponding to the pseudocubic axes. (**c**–**e**) Crystal models of pseudocubic unit cells for LFO *c*, *a*, and *b*-type domains when viewed along *a*_*PC*_ axis of the substrate, with the top row representing + types and the bottom row representing − types. In *c* domains, La atoms displace in opposite directions along the viewing direction, forming elliptical La columns with the long axis of the ellipse indicated by arrows. In contrast, La atoms displays alternating left–right shifts of rows of atoms for *a* domains and up-down shifts of columns of atoms for *b* domains, as indicated by the arrows in (**d**) and (**e**).

Bulk LFO possesses an orthorhombic structure (space group: *Pbnm*) with lattice parameters *a* = 5.552 Å, *b* = 5.563 Å, and *c* = 7.843 Å, or equivalently, *a*_*PC*_ = *b*_*PC*_ = 3.930 Å, *c*_*PC*_ = 3.922 Å (where PC stands for pseudo cubic and *a*_*PC*_ = *b*_*PC*_ = $\sqrt {a^{2} + b^{2} } /2$, *c*_*PC*_ = $c/2$), as shown in Fig. 1a. The FeO_6_ octahedra tilt following the a^−^a^−^c^+^ pattern as described by Glazer notation^38^, and the La atoms are displaced from the face center.

When LFO is grown as a thin film on other perovskites, its *c*_*PC*_ axis can align with any of the substrate’s PC axes, giving rise to six potential domain configurations denoted as *a* ± , *b* ± , *c* ± . The alignment of crystallographic axes between each type of film domain and the substrate is illustrated Fig. 1b. Specifically *a*+, *b*+ and *c*+ domains are related by 90-degree rotational operations, while + and − types (e.g., *c*+ vs. *c−*) are related by mirror symmetry.

**Figure 2:**
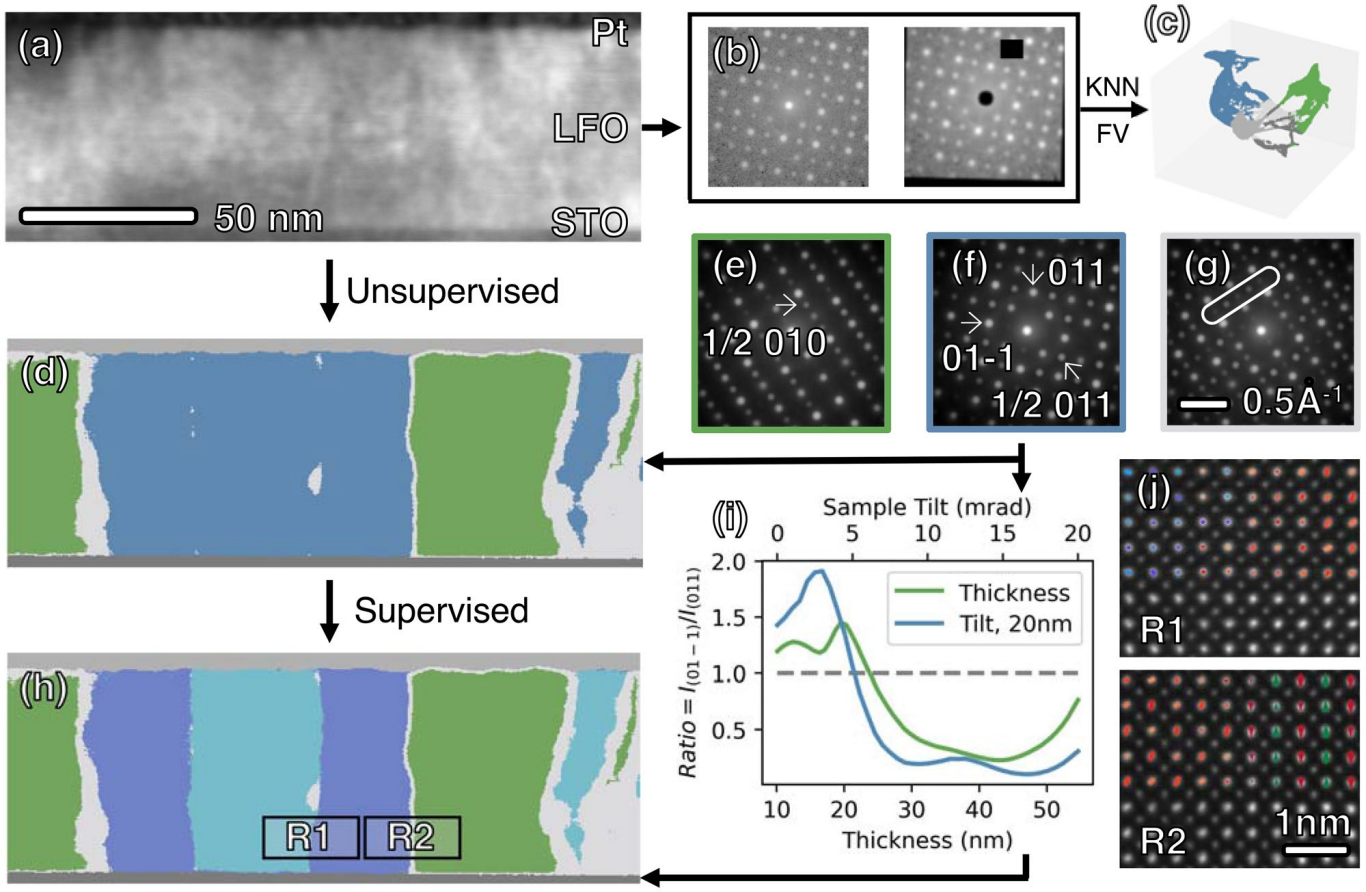
(**a**) Dark field image reconstructed from a 4D-STEM dataset consisting of 512 × 200 DPs. Unsupervised ML is first applied to cluster the given dataset with three major steps. First, two augmentations of diffraction pattern were generated through random affine transformation and masking of pixels, as shown in (**b**). Then, a ResNet-18 network was trained to maximize similarity between the two augmentations, thereby extracting a 1 × 512 feature vector (FV) for each diffraction pattern. The FVs were then partitioned into five clusters with KMeans, as depicted in (**d**) with 5 colors, and the corresponding manifold structure is shown in (**c**). The determined domain types are then mapped back in to real space as shown in (**d**). The top and bottom gray layers represent Pt and STO, respectively. Within the region of the film, three phases are identified as green [*b* domains with corresponding DP in (**e**)], blue [*c* domains, DP in (**f**)], and gray [domain boundaries, DP in (**g**)]. In (**g**), unique reflections for both *b* and *c* domains are visible, such as those within the white box, possibly resulting from the overlapping of the two domains when they are inclined with respect to the electron beam direction. (**h**) The application of a supervised ML method as described in main text enabled the further differentiation of *c*+ and *c−* domains (represented by light and dark blue, respectively) from the *c* type domains [represented by blue in (**d**)]. (**i**) The intensity ratio between 01-1 and 011 reflections of a simulated diffraction pattern for the *c*+ domain changes as a function of sample thickness with no sample mistilt (green profile), and also as a function of sample mistilt with a constant 20 nm sample thickness (green). The inversion of intensity asymmetry occurs at the point where the profile crosses the gray dashed line, corresponding to an intensity ratio of 1. (**j**) Atomic resolution STEM images acquire from *c*± domains wall (R1) and *c*/*a* domains wall (R2) in (**h**). At R1, La columns appear elliptical (corresponding to Fig. 1c) with direction of long axis indicated by colors (red for + 45° and blue for − 45°). The change in direction of long axis corresponds to *c*± domains wall. In the right part of R2, columns of La atoms show alternating up-down shifts indicated by red and green arrows, corresponding to *b* domains (model in Fig. 1f).

When observing along the *a*_*PC*_ axis of the substrate, distinct domains showcase unique projected atomic structures, as illustrated in Fig. 1c–e. These differences also manifest in DPs, allowing for their differentiation through STEM. For instance, in the case of *b*± domains, antiparallel displacements of La in a ↑↓ pattern with a periodicity of two (Fig. 1e) result in the doubling of unit cell along <010>_pc_, leading to the appearance of ½ 010_PC_ superlattice reflections in their DP (Fig. 2e). On the contrary, DPs of *c*± domains (Fig. 2f) do not display the same reflections but rather exhibit ½ 011 reflections, reflecting the doubling of unit cell along <011>_pc_ (Fig. 1c).

Furthermore, the + and − variants of each domain types display similar DPs but with opposite intensity asymmetries. Similar to the breakdown of Friedel’s law in ferroelectric perovskites^13^, antiparallel displacements of La also leads to intensity differences of Friedel’s pairs, $I_{{\vec{G}}}$ and $I_{{\mathop{G}\limits^{\leftarrow} }}$. For instance, in the case of *c*-type domains in Fig. 2f, such intensity asymmetry can be quantified from 011 and 01-1 reflections. This characteristic allows for the differentiation between mirrored domains based on the intensity of their respective DPs.

Nevertheless, it is crucial to account for potential experimental uncertainties. Factors like sample thickness and mistilt can introduce variations in intensity asymmetry, raising the possibility of inaccurate domain determinations. For instance, simulation results for *c*+ domains in Fig. 2i demonstrate a reversal in intensity asymmetry between 011 and 01-1 reflections when the sample thickness changes from approximately 10 nm to about 22 nm. Furthermore, a similar reversal can occur at a constant thickness of 20 nm when the sample mistilt exceeds roughly 5 mrad. Given the inherent uncertainties in experimental scenarios, relying solely on intensity asymmetry of specific Friedel’s pairs for domain determination becomes less reliable.

Advancing beyond intensity comparison from individual Friedel’s pairs, ML emerges as a promising approach for DP analysis, revealing hidden information, such as sample thickness and strain states^29,30,32^, which conventional methods may overlook. By harnessing advanced pattern recognition and feature extraction techniques, ML approach provides a comprehensive evaluation of the entire 128 × 128-pixel DP. This ensures improved data efficiency and reliability in mapping domains. In our study, we investigate the potential of employing both unsupervised and supervised ML techniques to map crystallographic domains in LFO. This approach enhances robustness against experimental uncertainties, allowing for efficient mapping over a wider field of view and achieving greater statistical significance.

### Implementation of unsupervised ML

In the first part of our study, we investigated the application of unsupervised ML for domain mapping in LFO epitaxial film sputtered on SrTiO_3_ (STO) substrate. Conventional methods, such as virtual dark field imaging, often necessitate prior knowledge of the sample to select specific reflections unique to each domain (e.g., ½ 010 for *b*± domains). However, this can vary from sample to sample, posing challenges in generalizing 4D-STEM analysis, especially for materials with unexpected phases. In contrast, unsupervised ML offers a more versatile and unbiased approach, as it requires no training data or prior knowledge of the sample. Instead, it autonomously identifies patterns from the raw DPs, allowing for a more flexible and data-driven analysis. This capability becomes particularly valuable when dealing with complex and unexplored materials.

The process of domain mapping using unsupervised ML is presented in Fig. 2a–g. DPs were acquired with Themis Z microscope (details in Method) at each scanning positions in the region shown in Fig. 2a, forming a 4D dataset. Subsequently, three main steps were employed to map domain: extracting one feature vector (FV) from each DP (Fig. 2b), clustering of FVs (Fig. 2c), and visualizing domain structure (Fig. 2d–g).

The extraction of FVs is pivotal in condensing the intricate information within 128 × 128-pixel DPs. This minimizes biases arising from factors beyond the material's intrinsic structures during subsequent clustering. As seen in Fig. 2e–g, high-intensity diffraction spots, containing vital structural data, represent only a small portion of the DP. In contrast, background pixels are susceptible to external noise, potentially introducing biases during clustering. Moreover, microscope misalignments like beam tilt and astigmatism can introduce artifacts, including DP shifts or distortion, further complicating reliable clustering. Hence, the imperative for enhancing the reliability and accuracy of domain mapping lies in robust feature extraction, involving dimension reduction of raw data through preprocessing. Here, we investigate the potential of an unsupervised ML approach to address this need.

A SimSiam-based contrastive learning approach was applied to extract higher-level features of each DP^37^ (details in Method). During each training epoch, two augmented versions of each individual pattern were generated using random affine transformations and were masked off in zero beams and other random regions, as shown in Fig. 2b. Subsequently, a ResNet-18 network^39^ was trained to maximize the similarity between the two augmentations in feature space. This procedure effectively eliminated irrelevant information, such as random background intensity and diffraction shifts or distortions between patterns, while preserved essential features associated with crystal structure. Ultimately, this approach yielded a compact 1 × 512 FV for each DP, enhancing the reliability and accuracy of domain mapping.

Following the extraction of FVs, they were partitioned into five clusters using KMeans algorithm, with the number of clusters determined by the elbow method^40,41^. The distribution of clustering results is depicted in a low-dimensional manifold structure in Fig. 2c, where the manifold structure is the projection of FVs to 3D space for visualization. Each color in Fig. 2c represents a unique group of FVs that exhibit higher similarity to one another. Finally, as each FV (or equivalently, each DP) corresponds to one pixel (scanning position), the labels obtained from the clustering results can be mapped back to real space for a better understanding of the domain structures, as depicted in Fig. 2d.

Real-space visualization in Fig. 2d unveils five distinct regions identified by unsupervised ML. The top and bottom (light and dark) gray layers correspond to amorphous Pt and STO substrate, respectively. Within the film region, three phases emerge, each displaying its averaged DPs as illustrated in Fig. 2e–g. In addition to DPs corresponding to *b*± (Fig. 2e) and *c*± (Fig. 2f) domains, a third type of DP is observed, exhibiting unique reflections originating from both phases (within the box in Fig. 2g). This distinct phase appears at the boundary between *b*± and *c*± domains in the real-space map, suggesting that it is likely attributed to the overlapping of *b* and *c* domains. The inclination of the domain walls with respect to the electron beam path leads to electron scattering from both phases, contributing to the appearance of the third DP. The discovery of this unexpected phase underscores the advantages of ML over conventional methods in uncovering complex and unforeseen material features.

The initial round of clustering effectively distinguished DPs with distinct features, such as those from different materials and LFO *b* and *c* type domains. However, mirrored domains (+ vs − types) revealed their distinctions through a subtler feature, namely intensity asymmetry. A second round of clustering was attempted on the FVs corresponding to *c* domains, aiming to distinguish *c*+ and *c*− domains. However, the results (not shown here) were found to be more sensitive to sample tilt and/or thickness rather than the specific phase of interest. This can be attributed to the unsupervised nature of the contrastive learning approach, which inherently emphasizes the most prominent features in the data. Consequently, the clustering process may be influenced by factors not directly related to the crystallographic domains of interest, leading to inaccuracies in domain mapping. To overcome these challenges, we turn our attention to supervised learning approaches.

### Implementation of supervised ML

By leveraging labeled training data, supervised learning can guide the model to focus on specific features related to mirrored domains, allowing for a more targeted and precise identification. Specially, we employed muSTEM^42^ to simulate labelled DPs for the *c*+ and *c−* domains, incorporating different experimental conditions such as sample thickness, mistilt, and defocus (details in Method). The labelled dataset was then used to train a ResNet-18 network, enabling the model to learn more discriminative features specific to the phase of interest. As a result, the model successfully differentiate of *c*± domains from *c* type domains, as illustrated in Fig. 2h comparing to Fig. 2d. For validation and sanity checks, atomic resolution images are obtained from the determined domain boundaries (boxed region in Fig. 2h), as shown in Fig. 2j. The atomic arrangements clearly exhibited changes across the boundaries, in agreement with the predicted phase, further confirming the reliability and accuracy of domain mapping using ML-assisted analysis of 4D-STEM dataset.

### Biased formation of LFO domains on STO substrate

The closely matched crystal structure between LFO and STO facilitates the formation of domains. However, the specific types primarily result from the need to minimize elastic energy due to lattice mismatch. In the case of LFO/STO heterostructures, LFO has an orthorhombic cell with *a*_*PC*_ = *b*_*PC*_ = 3.930 Å, *c*_*PC*_ = 3.922 Å, while STO has a cubic unit cell with *a*_*PC*_ = *b*_*PC*_ = *c*_*PC*_ = 3.905 Å. Given that all three principal axes of LFO are longer than their counterparts in STO, the arrangement least favorable to minimizing compressive strain involves aligning the shortest axis *c*_*PC*_ perpendicular to the substrate, thereby precluding the observation of *a* ± domains. Conversely, when considering the equivalent in-plane principal axes of STO, the LFO *c*_*PC*_ axis have an equal chance to align with any of the four in-plane axes of STO (Fig. 1b). Consequently, both *c* and *b* domains may form in equal proportion, alongside mirrored domains represented by *c*+ and *c−* types, with equal proportions observed within the *c* domain groups (25% *c*+, 25% *c−*, 50% *b*).

**Figure 3:**
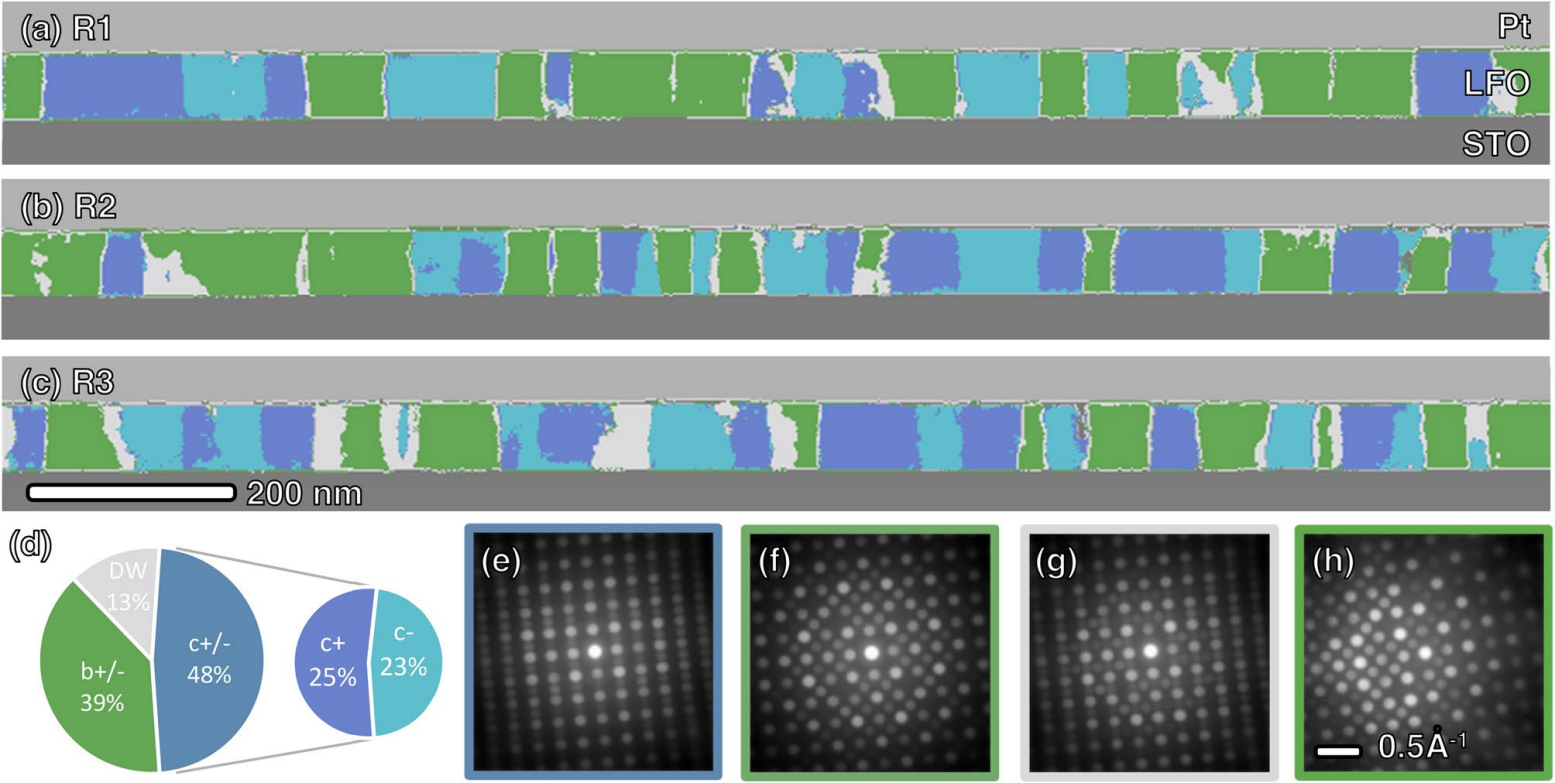
(**a**–**c**) Phase mapping from three TEM lamellas lifted-out from different regions of the sample. (**d**) Volume fraction of each phase extracted from three maps. (**e–g**) Average DP for *c*, *b*-type domain and domain walls respectively. (**h**) One representative DP with large sample mistilt.

As expected, domain mapping in Fig. 2h reveals both *c* and *b*-type domains but not *a*-type. To further validate this hypothesis, accounting for domain size and local inhomogeneity, a larger field of view was necessary. Thus, three TEM lamellas were extracted from various locations of the thin film sample, each yielding a 4D-STEM dataset comprising 80 × 1024 DPs. These datasets covered a total length of 1.5 µm. The datasets were acquired using uncorrected Titan STEM at a voltage of 300 kV and a semi-convergence angle of 1.25 mrad. Despite the presence of thickness gradient and substantial mistilt (> 5 mrad in Fig. 3h) due to the extended field of view, the ML method effectively clustered the DPs into different phases, as shown in Fig. 3a–c. The volume fraction of each phase is summarized in the pie chart in Fig. 3d. The results affirm the previously discussed hypothesis that particular domain types are preferred due to their ability to minimize elastic energy from lattice mismatch.

### Coarsening of domains at elevated temperature

Understanding the evolution of domain structures at elevated temperatures holds importance in deciphering the mechanisms governing domain formation and stability. Among the suggested mechanisms for domain formation is the phase transition that takes place during the cooling from the growth temperature to room temperature. In the case of LFO, it undergoes phase transformations upon heating from *Pbnm* to ${{R\overline{3}c}}$ and ${{Pm\overline{3}m}}$ at 1200 K and 1600 K, respectively^43^. As a result, annealing the film has the potential to reset the domain structure. To explore this hypothesis, in-situ experiments are performed using a DENs Solutions Wildfire heating holder to track the evolution of domain structures during heating.

**Figure 4:**
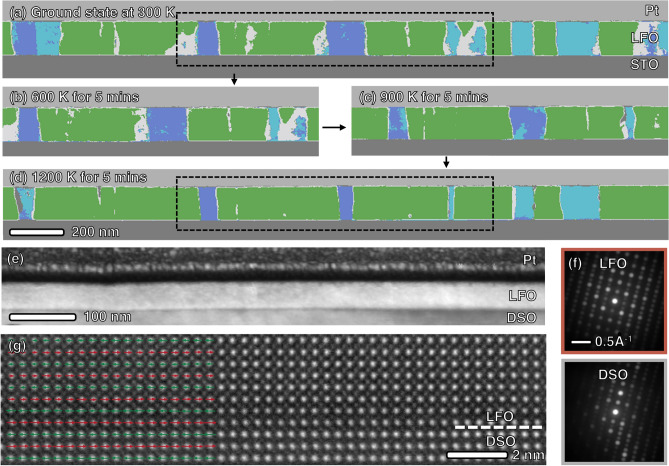
Phase mapping of the identical region at (**a**) ground state at 300 K, (**b**) after heat treatment at 600 K (**c**) 900 K and (**d**) at 1200 K for 5 min, respectively. Panel (**b**) and (**c**) corresponds to the boxed region of (**a**) and (**d**). (**e**) Dark field image reconstructed from a 4D-STEM dataset taken from LFO/DSO heterojunction. (**f**) Average DPs of LFO and DSO from the given dataset. (**g**) Atomic resolution STEM image from the LFO/DSO interface. The rows of La/Dy atomic columns show alternating left–right shifts as indicated with the red and green arrows. The displacement follows the same pattern dictated by the DSO substrate and corresponds to *a*-type domain (cf. Fig. 1d).

The domain maps obtained at 300 K after heat treatment at 600 K, 900 K, and 1200 K for ~ 5 min are depicted in Fig. 4b–d, revealing a coarsening of domains compared to the as-prepared sample in Fig. 4a. Notably, the regions identified as domain walls gradually disappear after heat treatment at 600 K and 900 K, with the most significant change occurring at 1200 K, which coincides with the *Pbnm* to ${{R\overline{3}c}}$ phase transition temperature. This observation suggests that the domains develop boundaries parallel to the electron beam direction rather than being inclined, likely due to the coarsening of domains along the TEM lamella thickness direction.

Furthermore, the original evenly distributed *b*/*c* domains (Fig. 4a) undergo a shift towards a *b*-type-favorable distribution (Fig. 4d) upon heat treatment. This shift is attributed to the broken in-plane lattice isotropy of STO due to the finite size of the TEM lamella. The lamella's dimensions, which are significantly smaller along the thickness direction (< 100 nm) compared to its length (> 10 µm), likely induce anisotropic expansion of the STO substrate during heating as comparing to isotropic expansion in bulk form. This anisotropic expansion leads to a more significant increase in the lattice parameter along the lamella's thickness direction, breaking the energy degeneracy between *b*/*c* domains. Consequently, the LFO *c*_*PC*_ axis favors alignment along the lamella length direction, resulting in a higher prevalence of *b* domains.

### Control domain formation with substrate

The crystal symmetry of the substrate is another significant factor influencing domain structure. In the case of cubic-symmetric substrates like STO, the equivalent in-plane axes result in an equal distribution of *b*/*c* domains. However, for an orthorhombic perovskite such as DyScO_3_ (DSO, *a*_*PC*_ = *b*_*PC*_ = 3.951 Å, *c*_*PC*_ = 3.957 Å, a^−^a^−^c^+^ tilting pattern), it is expected that the degeneracy is lifted, leading to an imbalanced domain distribution or even a monodomain. To investigate the impact of substrate crystal symmetry, LFO thin film was sputtered under the same conditions but on DSO (001) substrate, as shown in Fig. 4e. Despite the substantial in-plane rotation between the film and substrate, LFO exhibited a monodomain.

The averaged diffraction pattern over the entire film region in Fig. 4f corresponds to an *a*-type domain. Upon closer examination at the interface, as seen in Fig. 4g, atomic-resolution imaging revealed lateral displacements of the La columns, following the Dy displacement pattern of the substrate and aligning with the expected atomic arrangements of *a*-type domain (Fig. 1d). This configuration minimizes elastic energy caused by tensile strain by aligning the shortest axis of LFO (*c*_*PC*_) along out-of-plane direction. These findings underscore the critical role of crystal symmetry in determining domain structure.

## Summary and discussion

In this study, we explore the application of unsupervised and supervised ML to analyze 4D-STEM dataset for domain mapping, which demonstrates robustness against experimental uncertainties beyond conventional methods. In the first part, by extracting FVs and clustering them with unsupervised ML schemes, DPs with distinct features, such as those from different materials or 90-degree domains are effectively distinguished (*cf.* Fig. 2d).

While dark field imaging can potentially achieve similar results, the ML method possesses key advantages, notably its independence from prior knowledge to set up specific experimental conditions and its capacity to simultaneously handle multiple phases without the need for additional experimental setups. This versatility allows the ML approach to uncover unexpected phases, exemplified by the detection of overlapped domains in the film region of Fig. 2d (gray phase). Moreover, the application of unsupervised ML offers a more robust and unambiguous approach to phase partitioning.

However, the intensity asymmetry for mirrored (+/*−* type) domains is not picked up by the unsupervised ML algorithm, because sample thickness and tilt could change the intensity in a similar fashion but in a more dominant way. Therefore, a second supervised ML network is trained on labelled simulation DPs covering different experimental conditions, which successfully differentiates *c*+/*−* phase. For the training of supervised ML, it is necessary to generate synthetic data via simulation due to the difficulty in obtaining labelled experimental data. Ensuring that the simulated data is a good representation of experimental data is therefore crucial. In Fig. 2, collection was conducted using a probe-corrected system, resulting in data that closely resembles the simulated ones. Conversely, in Figs. 3 and 4, an uncorrected microscope was employed for data collection. This choice of instrumentation may lead to DPs that differed more from the simulations, contributing to higher levels of noise in the phase maps. Factors such as the larger scanning step size and convergence angle used in these experiments further contributed to the deviation from the ideal conditions used in simulation.

Overall, in this work, we employed ML algorithms to map the crystallographic domain structures in LFO films and apply this method to study the impact of temperature and substrate symmetry on domain formation. The result reveals the mechanism for domain formation and evolution. The methodology use in this work could also be readily generalized to other systems with complicated domain structure. For instance, ferroelectric materials have gained much attention, especially ones with complex phases such as polar vortices. Mapping these structures over a larger scale in STEM for statistical analysis can be challenging due to unknown experimental conditions. Since the polar displacement alters the intensity asymmetry of DPs in a similar fashion to the mirrored crystallographic domains, it is possible to train regression ML network to map out the polarization direction and magnitude.

## Materials and methods

### Epitaxial thin-film growth

LaFeO_3_ films of high crystalline quality were grown on both TiO_2_ terminated SrTiO_3_(100) substrates and solvent washed DyScO_3_(001) substrates by way of off-axis magnetron sputtering^44^. SrTiO_3_(100) substrates were prepared by soaking in DI H_2_O at 80 °C for 5 min, then soaked in buffer Hydrofluoric acid for 28 s, and rinsed in DI H_2_O. SrTiO_3_ substrates were then annealed at 1050 °C for 2 h for better terrace formation. DyScO_3_(001) substrates were soaked in Acetone for 5 min and Isopropanol for 3 min with light sonication, and then rinsed in DI H_2_O. No annealing was done to DyScO_3_ substrates. Films were grown in a temperature range between 400 and 500 °C, with a 5% O2 and Ar gas mixture, and at a rate of 1nim/min.

### Scanning transmission electron microscopy

TEM samples were produced following standard lifting-out procedure using the FEI Helios NanoLab 600 FIB/SEM system. Thinning to electron transparency was achieved by progressively reducing the energy of the Ga beam, starting from 30 kV down to 5 kV.

Four-dimensional scanning transmission electron microscopy (4D-STEM) datasets were acquired using Electron Microscopy Pixel Array Detector (EMPAD). Data presented in Fig. 2 was collected with a probe-aberration corrected Thermo Fisher Scientific Themis Z G2 microscope, operating at an acceleration voltage of 300 kV and a semi-convergence angle of 0.5 mrad. Data presented in Figs. 3 and 4 was collected with an uncorrected Thermo Fisher Scientific Titan microscope at an acceleration voltage of 300 kV and a semi-convergence angle of 1.25 mrad.

### In operando heating

Sample for in operando heating (Fig. 4a–c) was prepared by lifting-out and transferring a cross-sectional lamella onto dedicated microelectromechanical systems (MEMS) chip using FEI Helios NanoLab 600 FIB/SEM system. The heating experiment was carried out with DENs solutions wildfire heating holder.

Throughout the experiment, the sample underwent heating cycle following the sequence: 300 K → 600 K (~ 5 min) → 300 K → 900 K (~ 5 min) → 300 K → 1200 K (~ 5 min) → 300 K. Data presented in Fig. 4a–c was acquired from the identical region of the sample at 300 K after the heating cycle at the corresponding temperature.

### Machine learning

Extraction of FVs for unsupervised ML was conducted following a SimSiam contrastive learning scheme. The code base is publicly available at https://github.com/PatrickHua/SimSiam. The task-specific augmentations used, as well as the FVs extracted for data presented in Fig. 2 can be accessed at 10.5281/zenodo.8349542. The SimSiam (Similarity-based Self-Supervised Learning) approach employs a dual neural network architecture, where the task of predicting a target representation from another representation (i.e., two different augmentations) of the same image fosters feature learning. In our study, diverse augmentations of the same DPs are generated through steps, such as random affine transformations simulating scan distortions and random masking of the zero beam, which carries minimal information about domain types. By prioritizing similarity maximization between the two augmented versions of each DP, the model effectively reduces the dimensionality of the DPs, emphasizing features most relevant to domain types. This approach proves instrumental in enhancing the efficiency of feature extraction while mitigating the impact of noise and irrelevant information.

Supervised ML involved training a neural network with a ResNET-18 structure^45^. The code base is publicly available at https://github.com/tding1/Neural-Collapse. To generate the training data, muSTEM was utilized to simulated DPs from *c*+ and *c−* domians, covering a range of simulation parameters. This parameter matrix included sample mistilt values ranging from 0 to 15 mrad in 1 mrad increments, sample thickness values from 10 to 75 nm in approximately 1.5 nm increments, and defocus values from −100 to 100 Å in 100 Å increments. Each simulated DP is paired with a categorical label denoting its corresponding domain type (i.e., 0 for *c*+ and 1 for *c−*). The neural network is structured to take an DP as input and make predictions on the domain type as output (0 vs. 1). Throughout each training epoch, the neural network iteratively conducts predictions on the domain types of all simulated DPs. Subsequently, its parameters are adjusted to minimize the dissonance between predictions and labels, iterated until the desired level of accuracy is attained.

## Data Availability

Experimental and simulation data are provided in the figures. Raw 4D-STEM data used for analysis in Fig. 2 are publicly available at 10.5281/zenodo.8349542. Additional data are available from the corresponding authors upon request.
