# Publisher Correction: Structural degeneracy and formation of crystallographic domains in epitaxial LaFeO_3_ films revealed by machine‑learning assisted 4D‑STEM

**DOI:** 10.1038/s41598-024-59454-0

**Published:** 2024-04-17

**Authors:** Menglin Zhu, Joseph Lanier, Jose Flores, Victor da Cruz Pinha Barbosa, Daniel Russell, Becky Haight, Patrick M. Woodward, Fengyuan Yang, Jinwoo Hwang

**Affiliations:** 1 Department of Materials Science and Engineering, Ohio State University, Columbus, OH 43210 USA; 2 Department of Physics, Ohio State University, Columbus, OH 43210 USA; 3 Department of Chemistry and Biochemistry, Ohio State University, Columbus, OH 43210 USA

Correction to: *Scientific Reports* 10.1038/s41598-024-54661-1, Published online 20 February 2024

The original version of this Article contained errors in Figure 2, where the text between panels (a)–(d)–(h), as well as the text between panels (b) and (c) did not display correctly. Between panels (a) and (d) it now reads ‘↓ Unsupervised’, and in between panel (d) and (h) it now reads ‘↓ Supervised’. Between panels (b) and (c) it now reads $\mathop{\longrightarrow}\limits_{{{\text{FV}}}}^{{{\text{KNN}}}}$.

The original Figure 2 and accompanying legend appear below.

**Figure 2:**
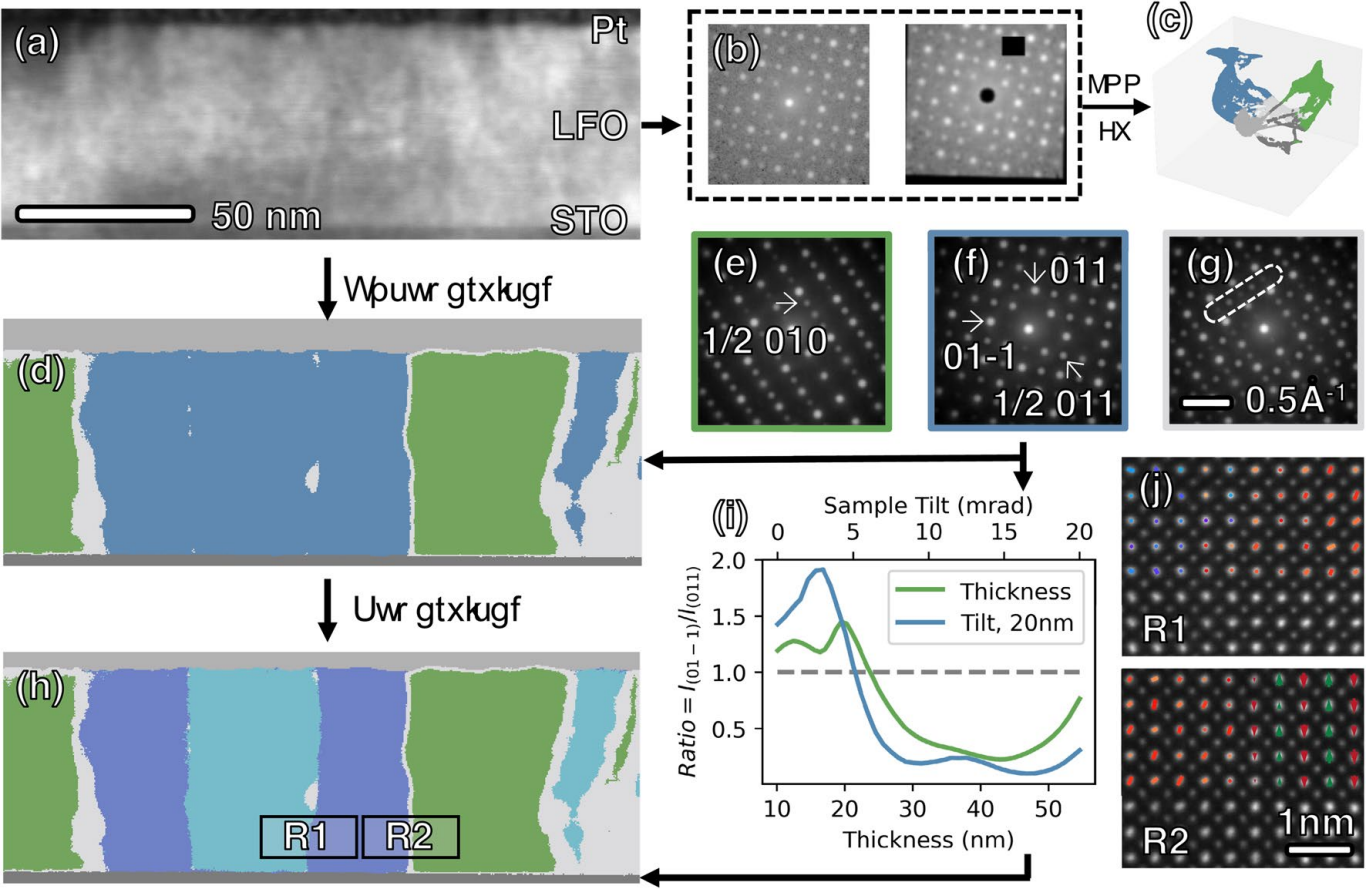
(**a**) Dark field image reconstructed from a 4D-STEM dataset consisting of 512 × 200 DPs. Unsupervised ML is first applied to cluster the given dataset with three major steps. First, two augmentations of diffraction pattern were generated through random affine transformation and masking of pixels, as shown in (**b**). Then, a ResNet-18 network was trained to maximize similarity between the two augmentations, thereby extracting a 1 × 512 feature vector (FV) for each diffraction pattern. The FVs were then partitioned into five clusters with KMeans, as depicted in (**d**) with 5 colors, and the corresponding manifold structure is shown in (**c**). The determined domain types are then mapped back in to real space as shown in (**d**). The top and bottom gray layers represent Pt and STO, respectively. Within the region of the film, three phases are identified as green [*b* domains with corresponding DP in (**e**)], blue [*c* domains, DP in (**f**)], and gray [domain boundaries, DP in (**g**)]. In (**g**), unique reflections for both *b* and *c* domains are visible, such as those within the white box, possibly resulting from the overlapping of the two domains when they are inclined with respect to the electron beam direction. (**h**) The application of a supervised ML method as described in main text enabled the further differentiation of *c*+ and *c*− domains (represented by light and dark blue, respectively) from the *c* type domains [represented by blue in (**d**)]. (**i**) The intensity ratio between 01-1 and 011 reflections of a simulated diffraction pattern for the *c*+ domain changes as a function of sample thickness with no sample mistilt (green profile), and also as a function of sample mistilt with a constant 20 nm sample thickness (green). The inversion of intensity asymmetry occurs at the point where the profile crosses the gray dashed line, corresponding to an intensity ratio of 1. (**j**) Atomic resolution STEM images acquire from *c*± domains wall (R1) and *c*/*a* domains wall (R2) in (**h**). At R1, La columns appear elliptical (corresponding to Fig. 1c) with direction of long axis indicated by colors (red for + 45° and blue for − 45°). The change in direction of long axis corresponds to *c*± domains wall. In the right part of R2, columns of La atoms show alternating up-down shifts indicated by red and green arrows, corresponding to *b* domains (model in Fig. 1f).

The original Article has been corrected.

